# Environmental Impacts and Behavioral Adaptations of Honeybees in Algeria: A Review of *Apis mellifera intermissa* and *Apis mellifera sahariensis* Characteristics

**DOI:** 10.3390/insects16060617

**Published:** 2025-06-11

**Authors:** Yamina Haider, Noureddine Adjlane, Nizar Haddad

**Affiliations:** 1 Department of Agronomy, Faculty of Sciences, M’hamed Bougara University, Boumerdes 35000, Algeria; 2 Laboratory of Bioinformatics, Applied Microbiology, and Biomolecules (BMAB), University of Boumerdès, Independence Avenue, Boumerdès 35000, Algeria; 3 Innovations & Business Development, Fresh Del Monte, Organic L’Ora, Amman 11814, Jordan; drnizar@yahoo.com

**Keywords:** *Apis mellifera*, adaptation, environmental conditions, abiotic stressors, biotic stressors, behavioural characteristics, Algeria

## Abstract

Honeybees are vital for pollination and the overall health of ecosystems. Since the 18th century, the intricate biology of honeybees has been a subject of scientific inquiry. Understanding their biological and behavioral characteristics is essential for effective beekeeping, honey production, and ecosystem sustainability. This review examines the environmental impact and management practices on the health of local honeybees in Algeria, focusing on *Apis mellifera intermissa* and *Apis mellifera sahariensis*. We summarize research findings on genetic diversity, morphometric traits, behavioral characteristics, and adaptation of local honeybees. Additionally, we discuss the threats posed by abiotic and biotic stressors and highlight the importance of conservation and sustainable management. The reviewed studies indicate that environmental factors significantly influence the behavioral characteristics and adaptation of local honeybees. Notably, the hygienic behavior of *A. m. intermissa* contributes to their resistance against diseases and the *Varroa destructor* mite. Further research in these areas is important for enhancing our understanding of honeybee health and population dynamics in Algeria, thereby informing strategies for sustainable beekeeping practices.

## Simple Summary

Honeybees are essential for pollinating plants and maintaining ecosystems. This review focuses on two subspecies in Algeria, *Apis mellifera intermissa* and *Apis mellifera sahariensis*, examining how they adapt to environmental challenges like climate and disease. We highlight the role of *Apis mellifera intermissa*’s natural behaviors in resisting pests like the *Varroa* mite. Understanding the unique traits of these local bees is key for sustainable beekeeping and protecting the biodiversity they support, which is critical for food production and ecosystem health.

## 1. Introduction

Honeybees play an essential role in pollination and maintaining ecosystems, contributing to biodiversity and agricultural productivity [1]. Understanding their behaviors is crucial for effective colony management, as these behaviors influence health, productivity, and resilience. Key behaviors such as foraging, thermoregulation, hygienic behavior, and grooming significantly affect colony stability and adaptability to environmental challenges. These behaviors are vital for managing pests and diseases, optimizing honey production, and ensuring sustainable beekeeping practices.

**Figure 1:**
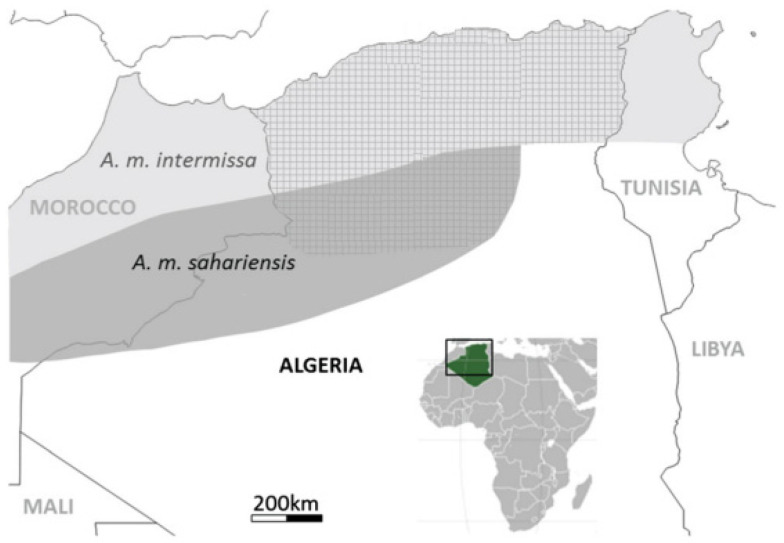
Distribution Map of Local Honeybees in Algeria (*A. m. intermissa* and *A. m. sahariensis*). The square frame highlights the geographical focus on Algeria, and the green area on the inset map shows Algeria's location within the African continent. The distribution boundaries of the honeybee subspecies are based on Adjlane et al. [12] and previous studies [4,13,14].

In Algeria, two subspecies of honeybees, *Apis mellifera intermissa* and *Apis mellifera sahariensis* hold particular significance due to their adaptations to the country’s diverse climatic zones. Algeria spans from a Mediterranean climate in the north [2] to arid and semi-arid regions with hyper-saline zones in the south [3]. *A. m. intermissa* is primarily found in North Africa, including Algeria and Tunisia (Figure 1), where it has adapted to the North African climate [4]. In contrast, *A. m. sahariensis* thrives in the harsh conditions of the Sahara Desert, with its range in Algeria including regions such as Béchar, Djebel Antar, and Beni Ounif [5].

Grooming behavior is known to influence honeybee resistance to pests like *Varroa destructor* and is shaped by genetic factors and natural selection [6]. However, grooming is just one aspect of their complex behavioral repertoire. Other key behaviors, including foraging efficiency, thermoregulation, and hygienic traits, are equally critical for colony survival and adaptation. Despite their ecological and economic importance, studies investigating the molecular and biochemical mechanisms underlying these behaviors in Algerian honeybees remain limited [7,8].

Conserving the genetic diversity of *A. m. intermissa* and *A. m. sahariensis* is vital due to their unique adaptations and increasing threats from habitat loss and climate change [9]. The recent availability of the western honeybee genome and transcriptome has provided valuable tools to explore the genetics of these subspecies, offering insights into their adaptation mechanisms [10,11].

This review synthesizes current knowledge about the behavioral characteristics and adaptations of Algerian honeybees, focusing on their role in sustaining local ecosystems and agriculture. By examining these subspecies’ behaviors, the study seeks to contribute to the development of sustainable beekeeping practices and conservation strategies that ensure the long-term survival of Algeria’s honeybee populations. Conserving local honeybee populations is critical for maintaining biodiversity and ecosystem services, which are essential for environmental stability and agricultural sustainability [8].

## 2. Materials and Methods

### 2.1. Literature Review and Data Collection

A systematic literature review was conducted to gather publications on the behavior and adaptation of local honeybee subspecies in Algeria: *Apis mellifera intermissa* and *Apis mellifera sahariensis*. The search covered the period from 1916 to 2024, including studies in all languages. After removing duplicates, 85 articles were filtered by titles and abstracts, and 30 studies were selected after a full-text review.

### 2.2. Inclusion Criteria

Inclusion criteria included studies on the behavioral, physiological characteristics, and adaptation of these subspecies. Articles not meeting these criteria were excluded.

### 2.3. Data Synthesis and Analysis

A thematic analysis was used to organize the study results, categorizing behaviors, environmental adaptation, and disease resistance. A qualitative synthesis was preferred due to the diversity of methodologies used in the studies.

### 2.4. PRISMA Flowchart

A PRISMA flowchart was included to illustrate the study-selection process, from identification to the inclusion of studies.

## 3. Results

The literature search identified a total of 85 papers related to our study. From this search, we finally gathered 30 studies, a summary of variables studied, and methods used in studies on the behavior and adaptation of local Algerian honeybees according to the methodology used and subspecies of honeybees (Table 1), and from each one we harvested information on factors influencing adaptation processes of local honeybees in Algeria (abiotic stressors and biotic stressors), biological characteristics of local honeybees in Algeria; *A. m. intermissa* and *A. m. sahariensis*: general population structure, biometric analysis, morphological differences. As well, information about adaptation to specific climatic conditions, temperature, and to diseases, and natural selection were also included.

## 4. Discussion

The 30 selected papers provided valuable insights into the stressors influencing adaptive responses in local honeybees in Algeria, specifically focusing on abiotic and biotic stressors, biological characteristics, and the adaptation mechanisms of *A. m. intermissa* and *A. m. sahariensis*. However, it is important to emphasize that the relatively small number of studies on these North African subspecies clearly indicates that they are significantly understudied compared to Central European subspecies such as *A. m. carnica* and *A. m. ligustica*. A simple search on Google scholar “*intermissa*” OR “*sahariensis*” gives about 7870 studies, and for “*ligustica*” OR “*carnica*” about 33,600 studies. This lack of research highlights the need for further investigations into the unique adaptations and ecological roles of *A. m. intermissa* and *A. m. sahariensis*, particularly in response to specific climatic conditions, temperature variations, diseases, and natural selection in their local environments.

### 4.1. Factors Influencing Adaptation Processes on Local Honeybees in Algeria

**Figure 2:**
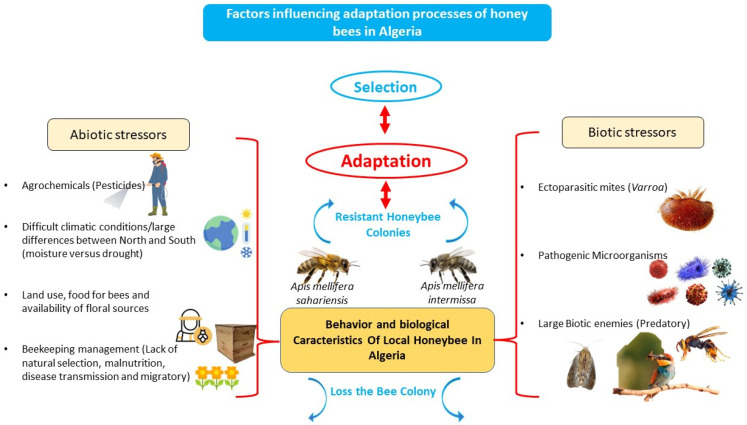
Schematic description of factors influencing adaptation processes on local honeybees in Algeria.

Lin et al. [37] focus on the potential impacts of biotic and abiotic stressors on honeybee physiology and colony health. Several factors influencing adaptation processes of local honeybees in Algeria (Figure 2). Adjlane et al. [25] highlight the threats to local bee populations in Algeria, including bee diseases, insecticide poisoning, ecosystem degradation, and climate change.

#### 4.1.1. Biotic Stressors

a. Ectoparasitic mites (*Varroa*): *V. destructor* feeds on both the hemolymph and the fat body of its honeybee host, consuming nearly a microliter daily [38,39,40] and significantly interfering with honeybee physiology [41,42]. The life cycle of *V. destructor* consists of two stages: the phoretic dispersal phase, during which it parasitizes adult bees, and the reproductive phase, closely synchronized with the host’s development, during which it infests immature individuals [43]. Bee diseases, mainly represented by the *V. destructor* and bee poisoning by insecticides, threaten the survival of bee colonies in Algeria [25]. The impact of the parasitic mite *V. destructor* increases the risk of bee colony collapse and is influenced by local environmental factors such as temperature and humidity [44].
b. Pathogenic microorganisms: The specific pathogenic microorganisms that cause biotic stress in honeybees include *Mellisococcus plutonius*, which is associated with European foulbrood (EFB) [45]. Other bacterial pathogens include the causative agents of American foulbrood (AFB), which are widely distributed and highly infectious [46]. Additionally, the fungal disease chalkbrood (CBD) affects honeybee broods [47]. *Nosema ceranae* is a microsporidian pathogen that has been identified as a cause of disease in honeybees [48]. These pathogens, along with other factors such as acaricide accumulation and unusual climatic conditions, contribute to the poor health status and vulnerability of honeybee colonies [49]. The development and progression of honeybee colonies are significantly influenced by various viruses, which pose a major threat to their health and well-being [50]. Viruses such as Deformed wing virus (DWV), Acute bee paralysis virus (ABPV), and Black Queen cell virus (BQCV) have been identified as having direct or indirect effects on individual bees and colony health [51]. *Varroa* exacerbates these effects by serving as a vector for these viruses, altering their transmission routes and potentially increasing their virulence [52]. Interestingly, while *Varroa* is associated with the spread of RNA viruses, it has been observed that the presence of certain viruses like SBV and BQCV can lead to a reduction in DWV viral titers, suggesting a complex interaction among viruses within honeybee colonies [52]. Moreover, the presence of *Varroa* mites and the associated viruses does not necessarily result in increased mortality of bee queens during the rearing process, indicating that the impact of viruses may vary depending on the context and the stage of bee development [53].
c. Large biotic enemies: Honeybees face various biotic enemies during nesting and foraging, including predators such as the Asian hornet (*Vespa velutina*) and Asian giant hornet (*Vespa mandarinia*), as well as pests like wax moths (*Galleria mellonella and Achroia grisella*), small hive beetles (*Aethina tumida*), and opportunistic predators like praying mantises (*Mantodea*) and frogs (*Batrachia*). The Asian giant hornet (*V. mandarinia*), native to Asia, invades hives, causing forager homing failure and colony paralysis, significantly reducing *A. mellifera* survival rates [54,55,56,57]. Similarly, the Asian hornet (*V. velutina*) preys on *A. mellifera* near hive entrances, disrupting foraging activity [54]. Wax moths (*G. mellonella* and *A. grisella*) and small hive beetles (*A. tumida*) damage hive structures, consume brood and honey, and cause colony collapse, particularly in weakened hives [54,57]. These biotic threats underscore the importance of implementing effective pest and predator management strategies to protect honeybee colonies.

#### 4.1.2. Abiotic Stressors

a. Climate change and habitat degradation: Climate change in Algeria has resulted in rising temperatures, increasing by 1.5 °C over 3 decades, and a 20% decline in precipitation in northern regions [4,21,58,59,60,61,62]. These shifts exacerbate drought, reduce floral resources, and extend the foraging season, placing stress on bee populations. Despite this, *A. m. intermissa* demonstrates resilience to high temperatures, foraging even at 40 °C, which aids colony survival during heatwaves. However, erratic rainfall impacts floral diversity, posing nutritional challenges for bees [21]. Conservation of floral diversity and breeding climate-resilient strains are vital to address these challenges [58,59]. Deforestation and habitat loss can also affect bee populations and their behavior and may lead to a decline in pollination services [59]. The economic value of bee pollination for crop production in Algeria is influenced by the number of visits and the aggregate effects of various bee species, including honeybees, carpenter bees, stingless bees, bumblebees, and solitary bees [59], The absence of national legislation and standards for Algerian honey could hinder the development of beekeeping in Algeria. Research on honeybee infections and available treatment options in the country remains limited. There is also a lack of studies on the behavior, physiology, and evolution of honeybees in Algeria. Furthermore, the microbiota of Algerian honeybees and honey is still poorly understood [5,25,58,60].
b. Environmental Stressors: The environment plays a significant role in shaping the behavioral characteristics of *A. m. intermissa* in Algeria. Haddad et al. [60] highlights the adaptability of *A. m. intermissa* to varying climatic conditions and its cleaning behavior, which may be a response to pressures such as temperature fluctuations and parasite infestation. Additionally, Menail et al. [12] suggests that pathogen-host interactions in *A. m. intermissa* could be influenced by the bee’s ability to withstand higher temperatures, a trait that becomes increasingly relevant with global warming and the resultant habitat shifts. Contradictions or interesting facts emerge when considering the impact of environmental stressors, such as pathogens and insecticides, on *A. m. intermissa*. Menail et al. [21] reports the presence of various pathogens and a potential vector, *Megaselia scalaris*, which could influence bee behavior through disease pressure. Menail et al. [21] discusses the adverse effects of insecticides on bee health, including changes in hypopharyngeal gland development and survival, which could alter foraging behavior and colony maintenance activities. Certain types of pesticides have been found to affect the behavior and survival rates of honeybees. The combination of pesticides, such as imidacloprid, chlorpyrifos, and glyphosate, can produce synergistic changes in the flight ability and behavior of honeybees, resulting in a decrease in flying duration and distance [63]. Furthermore, worst-case environmental concentrations of pesticide mixtures have been shown to cause higher mortality rates and disturbances in biochemical markers in honeybees [64]. Pesticides, particularly those with neurotoxic properties, have been reported to impact the nervous systems of local bee subspecies in Algeria, such as *A. m. intermissa* and *A. m. sahariensis* [24]. In summary, environmental factors such as climate, pathogens, and anthropogenic stressors like insecticides, significantly influence the behavioral characteristics of *A. m. intermissa* in Algeria. Adaptations to high temperatures and cleaning behaviors are beneficial traits for coping with environmental challenges [21], while pathogen prevalence and insecticide exposure may induce stress responses that affect bee health and behavior [61,62]. Understanding these interactions is essential for the conservation and management of this important pollinator species.
c. Beekeepers’ management: According to Aglagane et al. [7] *A. m. sahariensis* decreases with increasing human management (beekeepers) intensity and precipitation. This indicates that the level of human intervention and environmental conditions play a role in the genetic makeup of the honeybee populations. The study found that high rates of hybridization with *A. m. intermissa* jeopardize the genetic integrity of the Saharan honeybee. This hybridization is attributed to factors such as the modernization of the beekeeping sector, the importation of foreign queens, large-scale queen breeding, and the regular movement of colonies, which have heavily impacted the genetic pool of locally adapted subspecies and caused genetic pollution through introgression.

These studies collectively emphasize the importance of understanding and addressing the abiotic and biotic stressors faced by honeybees in Algeria, as they have significant implications for beekeeping, honey production, and overall agricultural health in the region.

### 4.2. Behaviour Characteristics of Local Honeybees in Algeria

#### 4.2.1. General Population Structure Stressors

There are at least 26 recognized subspecies of *Apis mellifera* [35]. These subspecies are grouped at the level of five evolutionary branches: A (African), C (Carnica), M (Mellifera), O (Oriental), and Y (Yemenetica) (Figure 1). Several studies have been characterized for the subspecies of *A. mellifera*, which are based on their morphological and genetic differences [26,34,65]. In some regions, by mixing several races, other subspecies belong to one or more evolutionary branches. Achou [26] found that Algerian honeybee populations consist of three different lineages: African, North Mediterranean, and West Mediterranean. They also identified a low level of genetic introgression from non-local honeybees, possibly due to the import of foreign honeybees. Loucif-Ayad et al. [65] confirmed the African origin of Algerian honeybee populations and identified two subspecies, *A. m. intermissa* and *A. m. sahariensis*. Bouzeraa et al. [9]. further supported the presence of African lineages in northeastern Algeria and noted higher genetic diversity in northern populations compared to southern populations.

Research has found evidence of genetic diversity and hybridization between different subspecies of honeybees in Algeria, particularly between *A. m. intermissa* and *A. m. sahariensis* [12]. This genetic diversity and hybridization may contribute to the adaptability and evolution of honeybees in response to their specific environmental conditions. Achou et al. [19] assessed the genetic analysis of Algerian honeybee populations revealed three evolutionary lineages: African (A), North Mediterranean (C), and West Mediterranean (M). The study identified eight different mtDNA haplotypes, with A1, A2, A8, A9, A10, and A13 belonging to the African lineage, while M4 and C7 were imported haplotypes. The pairwise *t*-test showed no significant difference between the two non-local haplotypes, M4 vs. C7. The local Algerian honeybees maintained a significantly higher presence, 96.9%, compared to non-local honeybees. This suggests that honeybee subspecies in Algeria have the potential to adapt and evolve in response to their specific environmental conditions. Furthermore, the study found evidence of hybridization between different subspecies, particularly between *A. m. intermissa* and *A. m. sahariensis* [6].

#### 4.2.2. Biometric Analysis

Bouzeraa et al. [20] conducted a morphometric study and found significant variations in various morphological traits among honeybees in the northeastern region of Algeria. Barour et al. [18,30] analysed the morphometric and forewing shape variations of *A. m. intermissa* in different regions of Algeria. They found distinct morpho clusters and shape differences between ecological regions, suggesting limited gene flow and possible anthropogenic introductions. A study by Bendjedid and Achou [27] discussed biometrics and performed statistical analyses on samples of bees from southern Algeria to define the position of this breed compared to others within *A. mellifica* from a morphological point of view. The description of the data by the univariate statistical method revealed that the bee of southern Algeria is small compared to that of Morocco, Tunisia, and northeastern Algeria for most morphological characters. The mean value of the yellow band width (coloring) of the two stations studied was 0.45 mm [66]. It was slightly higher than that given by Achou [66], which is of the order of 0.40 mm. This size differentiation was due to the low richness of the vegetation and the difficult climate of southern Algeria, which makes this bee have a lighter body to travel long distances in search of its food. We can attribute this differentiation to the existence of a north-south gradient for certain morphological characters [66]. Indeed, one of the first examples of the north-south gradient was provided for bees by a number of Russian authors, such as Chochlov [29], Michailov [67], Aplatov [68], and Ruttner and al [69], which found that the length of the tongue gradually decreased from north to south.

#### 4.2.3. Morphological Differences

**Figure 3:**
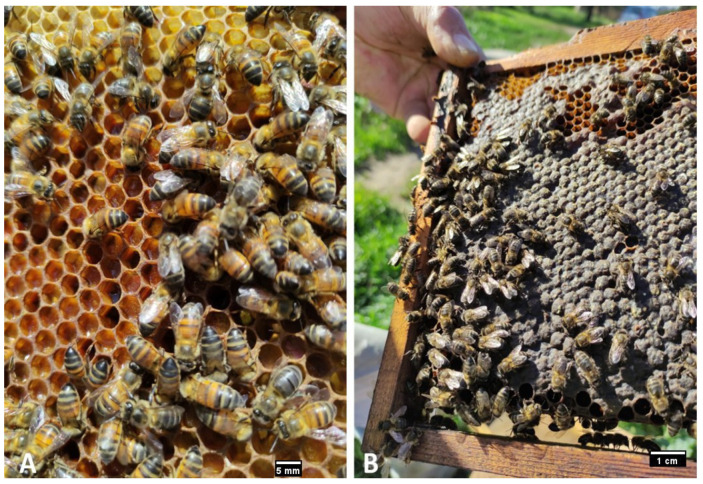
Original photos of the local honeybee in Algeria: (**A**) *A. m. sahariensis* (**B**) *A. m. intermissa*.

According to Bendjedid and Achou [27], the average value of the width of the yellow band (coloring) in the subspecie *A. m. sahariensis* shows a high dispersion, with a standard deviation ranging from 0.032 mm to 0.326 mm. Moreover, these results, concerning the coloring, confirm the descriptions left by the brother Adam [13] about the bee of southern Algeria. This author claims that a bee with yellow coat exists in very large numbers in this region. The same is true of the authors Garnery et al. [70], Franck et al. [14], and Loucif-Ayad [71], who confirm the existence of the yellow bee in southern Algeria (Figure 3).

One study focusing on *A. m. intermissa*, the native honeybee subspecies in Algeria and North Africa, found that these bees are distinctly darker in color with light illumination on the tergites [21]. This study also noted that the bees are small in in size compared to Algerian honeybees, specifically the subspecies *A. m. intermissa*, which are smaller in size compared to other subspecies [16]. This smaller size may be due to genetic factors specific to the Algerian population, as well as adaptations to the local environmental conditions. As for the Saharan bee *A. m. sahariensis*, it is characterized by its small size, yellow color, non-aggressive nature, and remarkable resistance to conditions difficult to heat and dry in the middle [36]. The queen, very long and large, is of yellow-red color going to red-cauldron, with the tip of the abdomen often dark, sometimes even black. This queen, very prolific, settles her spawning with a lot of economy; in spring she arrives, thanks to the sweetness of time, to lay beyond the possibilities of incubators [4].

#### 4.2.4. Nervousness and Aggressive Defense Behavior

Local honeybees in Algeria are known for their nervous and aggressive behavior when defending their hives [16]. Weller [36] discusses the existence of behavioral syndromes in individual honeybees, emphasizing the importance of considering both individual and colony-level personalities in understanding honeybee behavior. Lastly, Al-Etby [72] reviews the defense behavior of honeybee hives, highlighting factors such as colony strength, queen health, and the secretion of alarming pheromones that contribute to the aggressiveness of bees. The Tellian bee *A. m. intermissa* indicates a limited position by the races of Africa [32]. This bee is very aggressive, nervous, and characterized by a high tendency to swarm by several royal cells [73]. By comparing its genome with those of others under subspecies, the adaptive potential of *A. m. intermissa* is evolved for high temperatures and for resistance against pest infestations *V. destructor* [21]. This behavior is believed to be a result of their adaptation to the challenging environmental conditions of Algeria.

#### 4.2.5. Abundant Use of Propolis

Propolis, a resinous substance collected by bees from tree buds, is used for hive construction and defense. Algerian honeybees have been observed to use propolis abundantly in their hives, which may be a result of their need for extra protection in their environment [16]. *A. m. intermissa* exhibits strong defensive capabilities, notably through its abundant use of propolis. This behavior is likely influenced by a combination of environmental pressures, pathogen prevalence, and resource availability, highlighting the subspecies’ remarkable adaptability to Algeria’s unique ecological conditions [34].

#### 4.2.6. Foraging Behavior

One of the notable behavioral characteristics of local honeybees in Algeria is their foraging behavior. Studies have shown that Algerian honeybees exhibit specific foraging behaviors that are adapted to the local environment. They have been observed to have preferences for specific floral resources, such as certain plant species or nectar sources. Additionally, local honeybees in Algeria have been found to exhibit a high degree of resource efficiency during foraging, visiting multiple flowers within a short period to maximize their collection of nectar and pollen [16]. The Saharan bees forage very far away from their hive [4]. The foraging behavior of local honeybees in Algeria is influenced by several factors, including the availability of food sources, location, and the selected plant species in their foraging range. The morphological characteristics of honeybees may also play a role in their foraging behavior [23]. For example, a study by Abou-Shaara et al. found that the tongue length of Algerian honeybees is shorter compared to other subspecies. This may affect their ability to access nectar from deep floral structures and could explain their preference for certain plant species with more accessible nectar sources.

### 4.3. Adaptation to Specific Climatic Conditions, Temperature, and Diseases

#### 4.3.1. Tolerance to Environmental Conditions

The subspecies *A. m. sahariensis*, which predominates in the south of Algeria, is known for its adaptation to drought conditions, indicating a high level of tolerance to the challenging environmental conditions of the region [12]. A recent study by Khedidji et al. [8] discusses the influence of various factors, such as diet, subspecies, and age, on the development, physiology, and behavior of local honeybees in Algeria (*A. m. intermissa* et *A. m. sahariensis*). The study showed that the amount of protein in the hemolymph was influenced by the subspecies, with the subspecies *intermissa* having more hemolymphatic proteins than *sahariensis* when the same amount of pollen was consumed. This suggests that *intermissa* optimizes its protein diet much better than *sahariensis*. The study also demonstrated that the subspecies have evolved under different floral environments, possibly making them more adapted to digest specific pollen diets, with the diet used in the study potentially being more adapted to *intermissa.*

#### 4.3.2. Resistance Against *V. destructor*

In recent years, the study of honeybee behavior has shed light on two important traits that contribute to the resistance against *V. destructor*, a parasitic mite that poses a significant threat to honeybee populations [74]. This behavior, known as *Varroa* Sensitive Hygiene, is a heritable trait of *Apis mellifera* and can be incorporated into queen breeding programs as a sustainable strategy for controlling *V. destructor* [75]. Both *Varroa* Sensitive Hygiene and Suppression of Mite Reproduction are traits associated with the hygienic behavior of honeybees in response to *V. destructor* infestation [76].

The researchers observed that honeybees detected and removed diseased broods and *V. destructor* through uncapping and removal of infested cells. The timing of this hygienic behavior was found to be crucial in reducing the risk of disease transmission and improving colony fitness [22]. It was observed that honeybees displayed hygienic behavior promptly, removing diseased broods and *V. destructor* infested cells before further transmission of pathogens or pests could occur [77]. Another study focused on the behavioral characteristics of honeybees in Algeria and compared them to other social insects, such as ants and termites [78]. The study specifically investigated hygienic behavior in honeybees, which is an important form of social immunity for these insects [75]. The researchers aimed to understand the underlying behavioral mechanisms of hygienic behavior in honeybees and how it relates to diseases and parasites like *V. destructor*.

The first study by Adjlane and Haddad [76] on the behavioral characteristics of local honeybees in Algeria was conducted on 40 colonies of *A. m. intermissa*. The objective of the study was to evaluate the hygienic behavior of bees, which is an important factor in resistance to *V. destructor*, a parasitic mite that is a major threat to honeybees. The results of the study showed that local honeybees have a high hygienic behavior. The rate of removal of dead brood infested with *V. destructor* was 91.56% in spring and 83.55% in autumn. These results are higher than those reported for other honeybee races, suggesting that local honeybees are well adapted to the local environmental conditions and have a good resistance to *V. destructor*. The study on the grooming and removal behavior of *A. m. intermissa* in Tunisia against *V. jacobsoni* provides valuable insights into the resistance mechanisms of this subspecies. The results show that *A. m. intermissa* workers are highly effective at detecting and removing both artificially infested and freeze-killed brood, with removal rates of up to 75% and 97–99%, respectively. Additionally, the bees were observed to actively groom off *V. destructor* mites, with a large number of injured mites dropping from naturally infested colonies [33]. Overall, the studies from Tunisia and Algeria provide strong evidence that *A. m. intermissa* bees have evolved effective mechanisms to resist *V. destructor* mites.

The hygienic behavior of local honeybees in Algeria is influenced by environmental factors such as temperature, humidity, food availability, and exposure to pesticides [79,80,81,82]. Natural selection has led to the development of *V. destructor* resistance in honeybee populations in South Africa and in Africanized bees in South America. These populations showed a decrease in *V. destructor* numbers per hive and survived untreated after high initial *V. destructor* density and colony losses [83]. To develop programs for selecting honeybees resistant to *V. destructor* in Algeria, methods like natural selection, selective breeding, and artificial insemination can be employed [17,31,79]. Conservation and sustainable management of local honeybee can be achieved through protecting their habitat, educating beekeepers, and supporting them [84,85]. By implementing these measures, beekeepers can contribute to the preservation and sustainability of local honeybee populations in Algeria.

## 5. Conclusions

The study of behavioral characteristics and adaptations of local honeybees in Algeria provides critical insights into their response to diverse environmental conditions. These findings can inform actionable strategies for beekeepers and policymakers, focusing on improving management practices, enhancing colony health, and supporting sustainable beekeeping initiatives.

Protecting the genetic diversity of local honeybee populations is crucial for maintaining pollination services, which are fundamental to agricultural productivity, ecosystem stability, and food security. Targeted breeding programs that prioritize resilience traits, such as disease resistance and environmental adaptability, can help reduce colony losses and improve productivity. Policymakers can support these efforts by implementing national standards and legislation for honey production, ensuring the sustainability and quality of the industry.

Further research into the genetic and environmental factors influencing biological traits in Algerian honeybees will aid in refining breeding and conservation strategies. Additionally, understanding the specific hygienic behaviors linked to *V. destructor* infestation and viral infections will provide beekeepers with practical tools to improve colony fitness and reduce disease prevalence.

By emphasizing sustainable practices and reducing genetic introgression, Algeria can strengthen its agricultural sector and contribute to global honeybee conservation. Policymakers and beekeepers alike must collaborate to prioritize these efforts, ensuring the long-term resilience of ecosystems and the critical pollination services they provide.

## Figures and Tables

**Table 1:** Summary of variables studied, methodologies applied, and subspecies investigated in research on the behavior and adaptation of local Algerian honeybees.

Paper	Subspecies of Honeybees	The Methodology Used/Year of Study/Sample Size
[15]	*A. m. intermissa*	Literature review, where the author analyzed existing research on honeybee aggression, synthesizing findings from various studies/1994.
[7]	*A. m. intermissa* *A. m. sahariensis* *A. m. carnica* *A.m. mellifera*	Microsatellite Genotyping: The study employed microsatellite markers to analyse the genetic variation of honeybees collected from 148 colonies across seven populations. Population Clustering: Researchers used statistical methods to group honeybee colonies based on their genetic similarities, revealing the presence of distinct populations. Genetic Structure Analysis [Fst]: This statistical test measured the level of genetic differentiation between honeybee populations separated by the High Atlas Mountains. Assignment Testing: The study used genetic data to determine the probability of individual bees belonging to the Saharan subspecies/2023.
[16]	*A. m. intermissa*	Beekeeper Survey: Researchers conducted a survey among beekeepers in various locations (Bejaia, Blida, etc.) to gather information about beekeeping practices and colony management. Morphometric Analysis: The study involved collecting and measuring 15 specific physical traits of worker bees (*n* = 445) sampled from 30 hives across different locations/1923. Statistical Analysis: The researchers used statistical methods to analyze the morphometric data and identify potential variations between bee populations.
[8]	*A. m. intermissa* *A. m. sahariensis*	Controlled Diets: The study reared emerging worker bees from both subspecies under controlled laboratory conditions, providing them with either a pollen-rich or pollen-deprived diet. Physiological Measurements: Researchers measured the survival rate, hemolymph protein content, hypopharyngeal gland development, and ovary development of the bees at three different ages (7, 14, and 21 days) for each diet and subspecies combination. Statistical Analysis/2022/100 to 120 honeybee workers.
[17]	*A. m. intermissa* *A. m. sahariensis*	Literature review, where the author analyzed existing research on the importance of environmental education, particularly among youth, to address honeybee decline and biodiversity loss/2020/.
[9]	*A. m. intermissa*	Sample Collection: Researchers collected honeybee worker samples (*n* = 30) from three locations within Jijel province, northeastern Algeria. Mitochondrial DNA Analysis: The study employed a technique called PCR-RFLP [Polymerase Chain Reaction-Restriction Fragment Length Polymorphism] to analyze the mtDNA COI-COII region of the collected bee samples. This technique identifies variations in the DNA sequence that can be linked to different evolutionary lineages. Data Analysis/2020.
[5]	*A. m. intermissa*	Bee Collection: Researchers likely collected bees from both resistant and susceptible colonies. Age Groups: The study separated bees into distinct age groups (4, 7, 15, and 21 days old) for analysis. Experience Levels: Within each age group, bees were likely categorized as experienced (exposed to mites previously] or naive no prior mite exposure). Mite Introduction: A standardized number of mites were introduced to individual bees in a controlled setting. Grooming Behavior Observation: Researchers observed and quantified the grooming behavior of each bee, measuring the number of mites successfully removed. Data Analysis/2020/600 honeybee workers.
[6]	*A. m. intermissa* *A. m. sahariensis*	Sample Collection: Researchers collected a large number of honeybees (*n* = 1286) from 12 provinces in northwestern Algeria. Geometric Morphometrics: The study employed a landmark-based geometric morphometrics approach. Statistical Analysis: The researchers used various statistical methods: Principal Component Analysis (PCA), Mahala Nobis Distance, Allometric Analysis, Cross-Validation PERMANOVA (Permutational Multivariate Analysis of Variance)/2021.
[18]	*A. m. intermissa*	Bee Collection: Researchers collected honeybee worker samples (*n* = 1655) from three locations within Jijel province, northeastern Algeria. Morphometric Measurements: wing size, leg length, body parts like head capsule width or inter-ommatidial distance (distance between eye facets)/2011.
[12]	*A. m. intermissa* *A. m. sahariensis*	Colony Health Monitoring: Researchers employed established methods to monitor honeybee colony health in various locations across northern and southern Algeria. This involves tracking colony losses over a specific period. Parasite and Pathogen Detection: The study used diagnostic techniques PCR to detect the presence and prevalence of common honeybee parasites in bee samples from the monitored colonies/2016/.
[19]	*A. m. intermissa* *A. m. sahariensis*	Sample Collection: Researchers collected a large number of honeybee worker samples (*n* = 582) from 22 regions across Algeria. Mitochondrial DNA Analysis: The study employed a technique called PCR-RFLP [Polymerase Chain Reaction-Restriction Fragment Length Polymorphism] to analyse the mtDNA COI-COII region of the collected bee samples. Data Analysis: The researchers analysed the RFLP patterns to determine the evolutionary lineages and mtDNA haplotypes present in each region/2015.
[20]	*A. m. intermissa* *A. m. sahariensis*	Sample Collection: Researchers collected honeybee worker samples (*n* = 414) from eight different locations across Algeria. Microsatellite Analysis: The study employed 14 microsatellite loci Population Genetics Analysis: The researchers used various statistical methods to analyse the genetic data: 1-Hardy-Weinberg Equilibrium Tests 2-Phylogenetic Analysis 3-Population Structure Analysis 4-Allelic Introgression Analysis/2016.
[21]	*A. m. intermissa*	Whole Genome Shotgun Sequencing: The researchers employed a technique called Whole Genome Shotgun (WGS) sequencing to generate a draft sequence of the entire *A. m. intermissa* genome. Genome Annotation: The study is currently in the process of annotating the draft genome sequence. This involves identifying and characterizing the genes and other functional elements within the DNA sequence. Comparative Genomics: The researchers plan to compare the *A. m. intermissa* genome sequence with the genomes of other honeybee subspecies/2020.
[22]	*A. m. intermissa*	Sample Size: The study involved 40 honeybee colonies of *A. m. intermissa.* Seasonality: The experiment was conducted in both spring and fall seasons to assess potential seasonal variations. Dead Brood Introduction: Researchers introduced a standardized number of dead brood cells into each colony and monitored their removal rate. Hygienic Behavior Measurement: The researchers calculated the percentage of dead brood removed by the bees within a specific time frame (removal rate) as a measure of hygienic behavior/2022.
[23]	*Apis mellifera*	The Reviewed Studies This section cannot be definitively described based solely on the resume of a review paper. However, the review analyzes findings from various studies that might have employed: − Behavioral Observations: Monitoring bee activity within and outside the hive to track foraging patterns and preferences. − Field Experiments: Manipulating factors like food source availability or flower types to observe how bees adjust their foraging behavior. − Chemical Analysis: Studying the composition of pollen and nectar collected by bees to understand their foraging choices. − Genetic Analysis: Investigating potential genetic variations between subspecies that might influence foraging behavior/2018.
[24]	*A. m. intermissa* *A. m. sahariensis*	Bee Sample: The researchers used adult worker bees for the experiment. Doses: Bees were exposed to various concentrations (doses) of thiamethoxam. Exposure Routes: The experiment involved two exposure routes: − Oral application − Contact application Replication: Each dose group included three cages with 20 bees each, and the entire experiment was repeated three times for robust data analysis. Mortality Monitoring: Researchers monitored bee mortality at regular intervals over a specific period (24 h in this case). Data Analysis: The study employed dose-mortality response. They calculated LD50 values. They also analysed the speed of mortality (kinetics) at different doses/2007.
[25]	*A. m. intermissa*	Beekeeper Survey: Researchers conducted interviews with beekeepers in mid-northern Algeria to gather information about colony health issues and potential causes of bee deaths. Information Gathering: The study has complemented the survey data with information from: − Beekeeping Cooperatives − Technical Institutes − Regional Veterinary Laboratories Data Analysis: The researchers analysed the combined data from various sources to identify potential factors contributing to colony mortality/2012.
[26]	*A. m. intermissa* *A. m. sahariensis* *A. m. iberiensis* *A. m. mellifera*	Sample Collection: Researchers collected samples from 663 honeybee colonies across six European and African subspecies. Wing Shape Analysis: The study employed geometric morphometrics, a technique that analyses the positions of specific landmarks [points] on the wing to capture the overall wing shape. Microsatellite Analysis: Researchers analysed the variations at six microsatellite loci within the nuclear DNA of the collected honeybee samples. Data Analysis: The study employed statistical methods to analyse both data sets: − Geometric Morphometrics − Microsatellite Analysis − Combined Analysis/2015/582 honeybee workers.
[27]	*A. m. intermissa*	Sample Collection: Researchers collected a large number of worker bees (*n* = 3400) from honeybee colonies across three ecological regions in northern Algeria. Geometric Morphometric Analysis: The study employed geometric morphometrics. This technique involves capturing the positions of specific landmarks (points) on the bee forewing and analyzing their spatial relationships. Statistical Analysis: The researchers used statistical methods to analyze the wing shape data: − Regional Variation − Colony-Level Variation − Genetic Influences − Environmental Adaptation/2014.
[28]	*Apis mellifera*	Sample Collection: Researchers collected worker bee samples from 317 colonies across five populations in Algeria. Mitochondrial DNA Analysis: The study analyzed the mtDNA sequences of the collected bees to identify different haplotypes. Microsatellite Analysis: The researchers genotyped the bees at 14 microsatellite loci Population Genetics Analysis: The study employed statistical methods to analyze the mtDNA and microsatellite data: Haplotype Distribution: Comparing the frequencies and distributions of mtDNA haplotypes across the five populations. Genetic Diversity: Measuring the genetic diversity within and between populations using microsatellite data. Africanization Level: Estimating the extent of Africanization by comparing the frequencies of African and European mtDNA haplotypes and microsatellite alleles/2023.
[29]	*A. m. intermissa* *A. m. sahariensis*	Sample Collection: Researchers collected honeybee samples from different locations in eastern Algeria along a north-south transect. Morphological Measurements: The study involved measuring 21 morphological traits on the collected bees. mtDNA Analysis: The researchers extracted and analysed mtDNA sequences from the bee samples to identify different haplotypes. Microsatellite Genotyping: The study involved genotyping the bees at 14 microsatellite loci using radioactive amplification. Population Genetics Analysis: The researchers employed statistical methods to analyse the mtDNA and microsatellite data: Pathological Analysis: The study involved monitoring bee colonies and identifying the causative agent of colony losses. Morphological Impact Biochemical Analysis/1916.
[30]	*A. m. intermissa*	Sample Collection: The study likely involved collecting worker honeybees from apiaries in northeastern and southern regions of Algeria Morphometric Measurements: Researchers measured various physical traits of the collected bees, including: Body size measurements (length, width), Wing characteristics (area, shape) Leg length Multivariate Analysis: The study employed two main statistical techniques for analysing the morphometric data: Principal Component Analysis (PCA) Linear Discriminant Analysis (LDA) Population Clustering: Based on the combined results from PCA and LDA, the researchers were able to identify three morpho clusters/2005.
[14]	*A. m. intermissa* *A. m. sahariensis*	Sample Collection: Researchers collected honeybee samples from a total of 738 colonies across 64 localities in Africa, Europe, and the Middle East. mtDNA Analysis: The study used DraI RFLP analysis of the COI-COII mtDNA region. Microsatellite Genotyping: The researchers genotyped bees from eight populations (Morocco, Guinea, Malawi, South Africa). Population Genetics Analysis: The study employed statistical methods to analyse the mtDNA and microsatellite data: mtDNA Lineage Distribution, Genetic Diversity Africanization Level: Estimating the extent of Africanization in European populations by comparing microsatellite allele frequencies. Population Differentiation: Comparing genetic variation between populations using various methods like Fst and Nei’s genetic distance (for both mtDNA and microsatellites)/2001.
[31]	*A. m. intermissa*	Honeybee Strains: The researchers established two honeybee strains through artificial selection: − High Pollen Hoarding: Colonies selectively bred for storing large quantities of pollen. − Low Pollen Hoarding: Colonies bred for storing minimal amounts of pollen. Selection Process: The selection involved choosing colonies with the highest and lowest pollen stores at the end of each season and using them as parents for the next generation. Data Collection: Pollen Stores Forager Behavior and Division of Labor Diurnal Foraging Patterns Brood Production Statistical Analysis: The study employed statistical methods to analyze the collected data: Pollen Hoarding, Colony-Leve, Components/1985.
[32]	major geographical races [subspecies] of *Apis mellifera*	The guide include: Clear and concise explanations of honeybee biology and behavior. Diagrams and illustrations of bee anatomy, life stages, and hive components. Practical advice on beekeeping techniques like hive management, swarm control, and honey harvesting. Information on selecting appropriate honeybee subspecies for specific beekeeping goals and local conditions/2002.
[33]	*A. m. intermissa*	Sample Collection: The study likely involved selecting honeybee colonies of *Apis mellifera intermissa* in Tunisia. Artificial Infestation Freeze-Killed Brood Natural Infestation Observations: Mite Removal. Mite Grooming. Mite Injury/1999.
[34]	*Apis mellifera*	Morphometric Analysis Statistical Analysis Behavioral Analysis: The passage briefly mentions including behavioral data alongside morphometrics for a more complete understanding of geographic variability/1988.
[35]	*A. m. intermissa*	33 characters were measured in each of 404 samples of honeybees from different regions and examined by multivariate analysis. The quantitative variation of characters, as correlated with the geographical distribution of bees, is shown in a graph/1978.
[4]	*A. m. sahariensis*	Personal Observations Comparative Analysis Field Trials/1960.
[36]	*A. m. intermissa* *A. m. mellifera*	Personal Observations Comparative Analysis Field Trials/2015.
